# TranscriptomeBrowser: A Powerful and Flexible Toolbox to Explore Productively the Transcriptional Landscape of the Gene Expression Omnibus Database

**DOI:** 10.1371/journal.pone.0004001

**Published:** 2008-12-23

**Authors:** Fabrice Lopez, Julien Textoris, Aurélie Bergon, Gilles Didier, Elisabeth Remy, Samuel Granjeaud, Jean Imbert, Catherine Nguyen, Denis Puthier

**Affiliations:** 1 Inserm U928, TAGC, Parc Scientifique de Luminy, Marseille, France; 2 Université de la Méditerranée, Marseille, France; 3 Institut de Mathématiques de Luminy, Campus de Luminy, Marseille, France; 4 ESIL, Université de Provence et de la Méditerranée, Marseille, France; 5 Service d'Anesthésie et de Réanimation, hôpital Nord - Assistance Publique, Hôpitaux de Marseille, Marseille, France; Harvard Medical School, United States of America

## Abstract

**Background:**

As public microarray repositories are constantly growing, we are facing the challenge of designing strategies to provide productive access to the available data.

**Methodology:**

We used a modified version of the Markov clustering algorithm to systematically extract clusters of co-regulated genes from hundreds of microarray datasets stored in the Gene Expression Omnibus database (n = 1,484). This approach led to the definition of 18,250 transcriptional signatures (TS) that were tested for functional enrichment using the DAVID knowledgebase. Over-representation of functional terms was found in a large proportion of these TS (84%). We developed a JAVA application, TBrowser that comes with an open plug-in architecture and whose interface implements a highly sophisticated search engine supporting several Boolean operators (http://tagc.univ-mrs.fr/tbrowser/). User can search and analyze TS containing a list of identifiers (gene symbols or AffyIDs) or associated with a set of functional terms.

**Conclusions/Significance:**

As proof of principle, TBrowser was used to define breast cancer cell specific genes and to detect chromosomal abnormalities in tumors. Finally, taking advantage of our large collection of transcriptional signatures, we constructed a comprehensive map that summarizes gene-gene co-regulations observed through all the experiments performed on HGU133A Affymetrix platform. We provide evidences that this map can extend our knowledge of cellular signaling pathways.

## Introduction

Microarray technology provides biologists with a powerful approach for comprehensive analyzes of cells or tissues at the transcriptional level. DNA chips are now widely used to assess the expression levels from all genes of a given organism. These data, most generally deposited in MIAME-compliant public databases, constitute an unprecedented source of knowledge for biologists [1]. As an example, until now, the Gene Expression Omnibus repository (GEO) host approximately 8,000 experiments encompassing about 200,000 biological samples analyzed using various high through-put technologies [2]. Consequently, this represents billions of measurements that reflect the biological states of cells or tissues recorded in physiological or pathological conditions or in response to various chemical compounds and/or natural molecules. As public repositories are continually expanding, we are facing the new challenge of designing new strategies to provide efficient and productive access to the available data.

To date, at least two major solutions have emerged. The first one applies a “*gene-centered perspective*”, as developed in the “GEO profile“ or “SOURCE” web interfaces [3]. This approach allows users to retrieve the expression profiles of a given gene in numerous curated experiments. Once a profile is selected, a list of similar profiles (*i.e.* neighbors) can be retrieved. Although GEO proposes several tools to refine the queries, cross-analysis through multiple experiments can not be performed. The second solution involves an “*experiment-centered perspective*” as developed in the “GEO DataSets” and “ArrayExpress” web interfaces [4]. This approach provides to biologists a set of classification tools to re-analyze selected experiments. Depending on the interface, supervised or unsupervised analysis (see below) can be pre-calculated or computed on demand. Again, as no meta-analysis tool is available, mining and compiling even few GEO Serie Experiments (GSE) remains a difficult and time-consuming task.

We therefore lack efficient tools allowing productive data mining of microarray databases. For example, querying whole public microarray data using a single gene identifier is an ambiguous procedure to extract relevant co-regulated genes. Indeed, depending of the biological context, genes can be involved in different signaling pathways and may be associated with different neighbors. As a consequence, combined queries should be more appropriate to build relevant gene networks. Moreover, numerous uninformative genes exist in microarray experiments. They correspond most generally to those with low standard deviation that are outside any natural gene cluster. These genes should be discarded from analysis as they are inevitably associated with false positive neighbors. These considerations motivated the present work and the development of a new approach that follows a “*transcriptional signature centered perspective*”. The goal was to build an application that would interact with a large database of transcriptional signatures and would implement efficient tools to analyze and visualize the results.

The first issue resided in the construction of a database containing high quality transcriptional signatures obtained in an automated fashion. Both supervised and unsupervised classification algorithms can be used in microarray data analysis [5]. Supervised methods aim at finding a set of genes whose expression profiles best correlate with a known phenotype. They provide a way to select informative genes by choosing the top k genes according to the results of a statistical test (*e.g*. Student's *t*-test, Significance Analysis of Microarrays, Signal to Noise Ratio, ANOVA) and by controlling the false discovery rate (FDR). In contrast, unsupervised classification approaches, achieve clustering of genes based on their respective expression profiles but are not intended to filter out uninformative genes. Some popular approaches in microarray analysis use either agglomerative methods (hierarchical clustering), partitioning methods (k-medoids, k-means, PAM, SOM, etc.) or methods aimed at capturing informative dimensions (PCA). A filtering step is most generally applied prior to unsupervised classification. One can select genes with high standard deviations, those displaying a proportion of values above a user-defined threshold or those having a given maximum (or minimum) value. However this procedure is extremely subjective and the number of selected genes may be over or under estimated. Finally, another limit of classical unsupervised methods also resides in their inability to accurately identify the actual number of clusters if no further argument is provided to the algorithm. As a consequence, additional algorithms for unsupervised classification have been proposed such as Quality Cluster algorithm (QT_Clust) [6], CHAMELEON [7] or Markov CLustering (MCL) [8]. However, none of them address both the filtering and partitioning issues. MCL is a graph partitioning algorithm whose ability to solve complex classification problems has been underlined in many applications including protein-protein interaction networks [9], sequence analysis (TRIBE-MCL) [10] or microarray analysis (geneMCL) [11]. In a graph representation of microarray data, nodes stand for genes and edges represent profile similarities between genes. As processing the full graph for partitioning is time-consuming and computer-intensive the geneMCL algorithm has to be run on a subset of genes that are selected using classical filters (*e.g.* high standard deviation or fold-change). As such a filtering procedure is not well suited for automated analysis of numerous experiments; we developed an adaptive density-based filter (DBF) whose goal is to isolate automatically informative genes from a dataset. Selected genes are next used to construct a graph that is subsequently partitioned using MCL. This modified version of MCL algorithm was termed DBF-MCL for “Density Based Filtering and Markov CLustering”.

In the present paper, we show that DBF-MCL provides very good results both on simulated and real datasets. The algorithm was run on 1,484 microarrays datasets (46,564 biological samples) performed on various Affymetrix platforms (human, mouse and rat). This led to the identification of 18,250 transcriptional signatures (TS) whose corresponding gene lists were tested for an enrichment in terms derived from numerous ontologies or curated databases using the DAVID knowledgebase [12] (Gene Ontology, KEGG, BioCarta, Swiss-Prot, BBID, SMART, NIH Genetic Association DB, COG/KOG, etc.) (see Figure S1 for an overview of the data processing pipeline). Informations related to biological samples, experiments, TS composition, TS associated expression values and TS keyword enrichment scores were stored in a relational database. A Java application, TBrowser (TranscriptomeBrowser), was developed and deployed using Java Web Start technology. Combined queries that can be done with an extended set of Boolean operators allow user to rapidly select sets of TS containing (or not) a given list of gene symbols. Based on these TS, a list of frequently observed neighbors can be created. As each TS is linked to a set of biological keywords (derived from ontologies), user can also search for those enriched in genes involved in specific biological processes. We show that TBrowser can be used to mine productively hundreds of experiments and to reveal underlying gene networks. Furthermore, using this unprecedented collection of TS we built the first synthetic transcriptional map of all human microarray data performed on Affymetrix HG-U133A platform and currently available in the GEO database.

## Results

### DBF-MCL algorithm

Conventional algorithms used for unsupervised classifications of gene expression profiles suffer from two main limitations. First, they do not filter out uninformative profiles and second, they are not able to find out the actual number of natural clusters in a microarray dataset. We can considerer genes as points located in a hyperspace whose number of axes would be equal to the number of biological samples. As it is difficult to perceive high-dimensional spaces, a common way to illustrate classification methods is to use a 2D representation. In Supplemental Figure S2, each point represents a gene and we are interested in isolating dense regions as they are populated with genes that display weak distances to their nearest neighbors (*i.e.* strong profile similarities). To isolate these regions we can compute, for each gene, the distance with its k_th_ nearest neighbor (DKNN). If k is relatively small, DKNN should be smaller for all genes falling in a dense area. Thus, the filtering procedure used in DBF-MCL starts by computing a gene-gene distance matrix D. Then, for each gene, DBF-MCL computes its associated DKNN value (with k being set typically to 100 for microarrays containing 10 to 50k elements). Distributions of DKNN values observed with both an artificial and a real dataset (Complex9RN200 and GSE1456 respectively, see thereafter for a description) are shown in Figure S3A and S3B (solid curve). The asymmetrical shape of the distribution observed in Figure S3B suggests the presence of a particular structure within the GSE1456 microarray dataset. Indeed, the long tail that corresponds to low DKNN values could indicate the existence of dense regions. The fact that regions of heterogeneous densities exist in the Complex9RN200 artificial dataset is even clearer as a bimodal distribution is observed. Next, we would like to define a critical DKNN value below which a gene can be considered as belonging to a dense area and that would depend on the intrinsic structure of the dataset. To this end, DBF-MCL computes simulated DKNN values by using an empirical randomization procedure. Given a dataset containing n genes and p samples, a simulated DKNN value is obtained by sampling n distance values from the gene-gene distance matrix D and by extracting the k_th_-smallest value. This procedure is repeated n times to obtain a set of simulated DKNN values S*_i_*. As shown in Figure S3 (dotted line), the variance of the simulated DKNN values is very low compare to that observed using the real dataset. Indeed, we can think of simulated DKNN values as the distances to the k_th_ element if no structure existed in the associated space. In this case, we would expect elements to be uniformly spread throughout the space and the variance of DKNN value to be low. In practice several sets S_1..q_ are computed and thus several distributions of simulated DKNN values are obtained. For each observed DKNN value d, a false discovery rate (FDR) value is estimated by dividing the mean number of simulated DKNN below d by the number of observed value below d. The critical value of DKNN is the one for which a user-defined FDR value (typically 10%) is observed. Given a set of selected genes, the next issue is to partition them into homogeneous clusters. This step is achieved through a graph partitioning procedure. In the created graph, edges are constructed between two genes (nodes) if one of them belongs to the k nearest neighbor of the other. Edges are weighted based on the respective coefficient of correlation (*i.e.*; similarity) and the graph obtained is partitioned using the Markov CLustering Algorithm (MCL).

### Performances of DBF-MCL on Complex9RN200 dataset

To test the performances of DBF-MCL algorithm we used a modified version of the complex9 dataset which was used earlier by Karypis *et al.* [7]. Since DBF-MCL is designed to handle noisy datasets, 200% of normally distributed random noise was added to the original data. The resulting dataset (which will be referred as Complex9RN200 thereafter, see Figure S4A and S4B) shows some difficulties for partitioning since it is composed of a noisy environment in which arbitrary geometric entities with various spacing have been placed. The two main parameters of DBF-MCL are k that controls the size of the neighborhood and the inflation I (range 1.1 to 5) which controls the way the underlying graph is partitioned. The effect of k on the selection of informative elements is shown in Figure S5A (Euclidean distance was used for this dataset). A steep ascending phase and a slow increasing phase (starting from a k values close to 40) were observed. This confirms the existence of areas with heterogeneous densities. In fact, the transition between the two phases reflects the transition from dense to sparse regions. Indeed, datasets produced with k values above 40 contain noisy elements (Fig S4C). In contrast, choosing k values in the ascending phase ensure the achievement of noise-free datasets. In the case of artificial data, satisfying partitioning results were obtained with inflation values close to 1.2 (Fig S4D–G) although in some cases some of the shapes were merged in a manner that appears to be meaningful (Fig S4E and S4G). We then compared DBF-MCL to several algorithms commonly used in microarray analysis. All of them were run multiple times with various parameters and the best solution was kept. In all cases, the Euclidean distance was used as a distance measure between elements. As these algorithms are not well-suited for noisy data, they were run on the 3,108 points extracted using DBF-MCL (k = 20). Also it is difficult to compare those algorithms to one another, some of them obviously failed to identify the shapes. Indeed, although k-means was run 10 times with random initial starts (and the right number of centers) it led to a very poor partitioning result (Fig S4J). Cluster Affinity Search Technique (CAST, Fig S4K) and Quality Cluster algorithm QT_CLUST (Fig S4I), gave also poor results as did the Self-Organizing Map (SOM) (data not shown). Hierarchical clustering was run with single linkage as arguments and the obtained dendrogram was then split into 9 clusters (Fig S4H). Patterns were well recognized using this method but prior knowledge of the number of clusters is a prerequisite. Thus both DBF-MCL and hierarchical clustering are able to properly identify complex shapes in a 2D space. The main benefit of using DBF-MCL resides in its ability to extract relevant informations from a noisy environment. However, a range of optimal values for inflation parameter needs to be defined to get the best results.

### Performances of DBF-MCL on GSE1456 dataset

**Figure 1:**
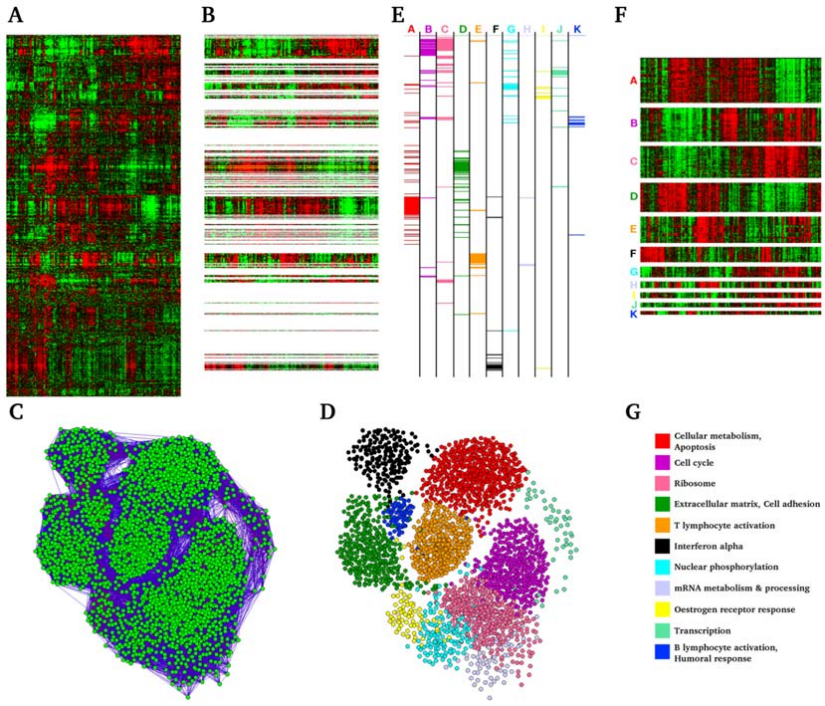
Results obtained with the GSE1456 dataset. DBF-MCL was run with GSE1456 as input (k = 100, FDR = 10%, S_1..3_, Inflation = 2). (A) Hierarchical clustering of the GSE1456 dataset. (B) Same as (A) but only informative genes are displayed. (C) The graph constructed with the 4,470 selected genes. (D) The graph after MCL partitioning. Each point is colored according to its associated class. (E) Correspondence between hierarchical clustering and DBF-MCL results. (F) TS obtained for GSE1456 (G) Functional enrichment associated with these TS.

Next, DBF-MCL was tested with microarray data to explore its effectiveness in finding clusters of co-regulated genes. To this end, we used the microarray data from Pawitan *et al.* [13], who studied gene expression profiles in a large cohort of Swedish patients affected by breast cancer. This experiment is recorded as GSE1456 in the GEO database. All sample (n = 159) have been hybridized onto the GPL96 platform (Affymetrix GeneChip Human Genome U133 Array Set, HG-U133A). The complete dataset (22,283 genes) was used for analysis. Figure S5B, shows the number of informative genes obtained with various k values. Again, two phases were observed suggesting that regions with heterogeneous densities exist in the GSE1456 dataset. As expected, the transition from dense to sparse regions was less marked than in the artificial dataset. A k value of 100 was chosen to allow the extraction of a large part of data that can be considered as noise-free. This value led to the selection of 4,470 elements out of the whole dataset (Fig. 1A–B). The graph partitioning procedure, using default MCL parameters (I = 2), generated 11 highly homogeneous clusters (Fig. 1C–F). As with the Complex9RN200 dataset, the results were very consistent with those obtained using hierarchical clustering although for some genes the clustering results differed (Fig. 1E). Importantly, partition results were not very sensitive to inflation values. Indeed, 10 and 12 clusters were observed with I set to 1.5 and 2.5 respectively (data not shown). All signatures were then submitted to functional enrichment analysis. A summary of the results is given in Figure 1G. As expected for a breast cancer dataset, TS were found to be related to (i) immune response (T-lymphocyte activation, B-lymphocyte activation and interferon alpha), (ii) primary metabolism (cell cycle, ribosome biogenesis, nuclear phosphorylation and transcription) which is probably reminiscent of tumor aggressiveness (iii), modification of local environment (extracellular matrix and cell adhesion) which could sign metastasis potential of each sample, (iv) and estrogen receptor status of breast tumors (estrogen response pathway). Altogether, these results underline the ability of DBF-MCL algorithm to find natural gene clusters within a randomly selected dataset. Indeed, for numerous additional microarray datasets hierarchical clustering results and DBF-MCL results were compared. As illustrated in Figure S5B for a representative set of experiments, setting k to 100 allows in all cases to delete noisy elements and to select only informative genes in a microarray dataset. Interestingly, in all cases meaningful partitioning results were obtained using inflation parameter set to 2.

### Systematic extraction of TS

**Figure 2:**
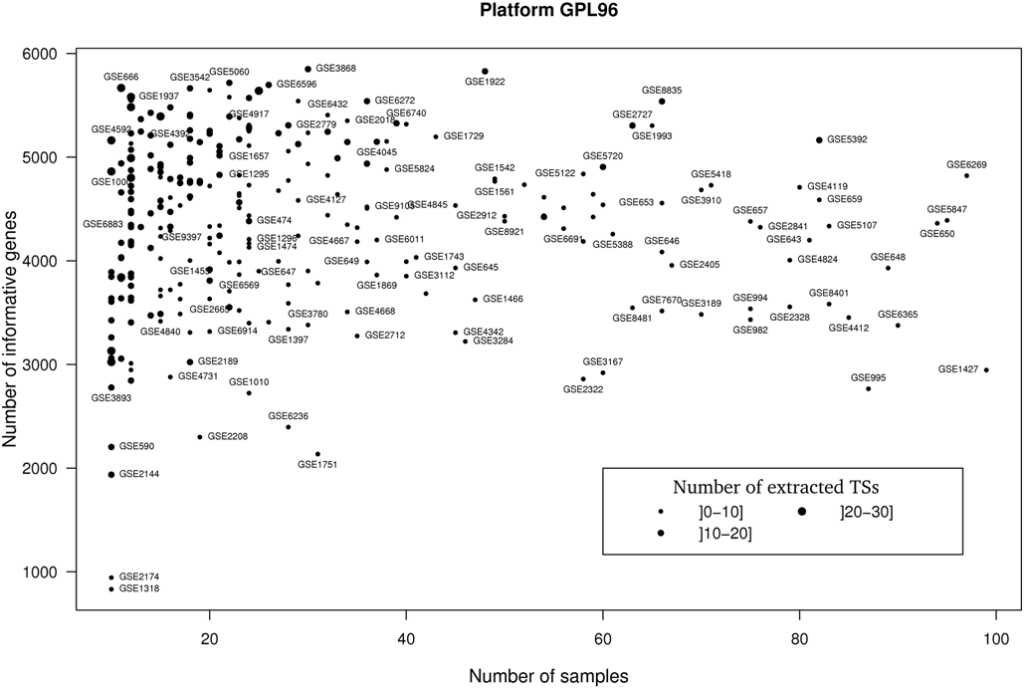
Large scale TS extraction from GPL96 experiments. DBF-MCL was run with default parameters (k = 100, FDR = 10%, S_1..3_, Inflation = 2). X axis corresponds to the number of samples in the experiment and Y axis to the number of informative genes. For each experiment, the number of associated TS is represented by the size of the dot. For clarity purpose only experiments with less than 100 samples are represented. Furthermore, the name of only some of them is displayed.

We next applied DBF-MCL algorithm to all experiments performed on human, mouse and rat Affymetrix microarrays and available in the GEO database (33 platforms, Supplementary Table S1 and S2). Only experiments containing more than 10 biological samples were kept for analysis. Overall, this dataset includes 46,564 biological samples hybridized in the context of 1,484 experiments. Each experiment was analyzed independently and subjected to TS discovery process (k = 100, FDR = 10%, S_1..3_, Inflation = 2). As mentioned in the Material and Methods section, we rank-transformed data from each biological sample to get a common input for DBF-MCL algorithm and to allow analysis of a large broad of experiments whose normalization status is frequently unknown. Furthermore, a distance based on Spearman's rank correlation coefficient was used for k_th_-nearest neighbor computation. This rank-based distance is known to be clearly more resistant to outlying data points than Pearson-based distance and thus ensured the selection of genes belonging to unmistakable clusters. The full pipeline was run on a server equipped with 6 CPUs and took about 4 days to complete. For the sake of clarity, only results obtained with GPL96 which is the most widely used Affymetrix microarray platform will be presented in this section (311 experiments related to GPL96 were analyzed, 12,752 hybridized samples). On average, 4,341 probes (min = 832, max = 5,849) per expression matrix were declared as informative by DBF-MCL suggesting that routinely 20% of the 22,283 probes measured on the HG-U133A array belong to a natural cluster. Graph partitioning generated on average 10.8 clusters (min = 2, max = 29) for each experiment and each cluster contained approximately 400 probes corresponding in average to 370 distinct gene symbols. Figure 2 shows a summary of these results. As expected no clear correlation was observed between the number of selected genes and the number of samples in the experiments which demonstrates the robustness of the filtering process. In contrast, a trend to produce more clusters in experiments containing few samples was observed. This was notably marked in experiments containing 10 to 15 samples. Such a bias is classical in data analysis. Indeed, if numerous values (*i.e*. samples) are used to estimate the expression profile of a given gene, outliers will have weak impact on distance calculation and the gene will be assign to the expected cluster. In contrast, when only few values are available, each of them has a greater impact on distance calculation. This results in producing more clusters with some of them having centers close to one another. This bias is also presumably amplified by the fact that small sample sets contain most generally a greater biological diversity compared to large sample sets as they contain fewer replicates. Overall, our analysis of GPL96 related experiments gave rise to 3,377 TS. The full analysis on the 33 Affymetrix platforms produced 18,250 TS which correspond to 220 millions of expression values. Partitioning results where manually checked for a large panel of experiments. Although, results seemed perfectible in few cases, they always appeared to be rational.

### The TBrowser interface

**Figure 3:**
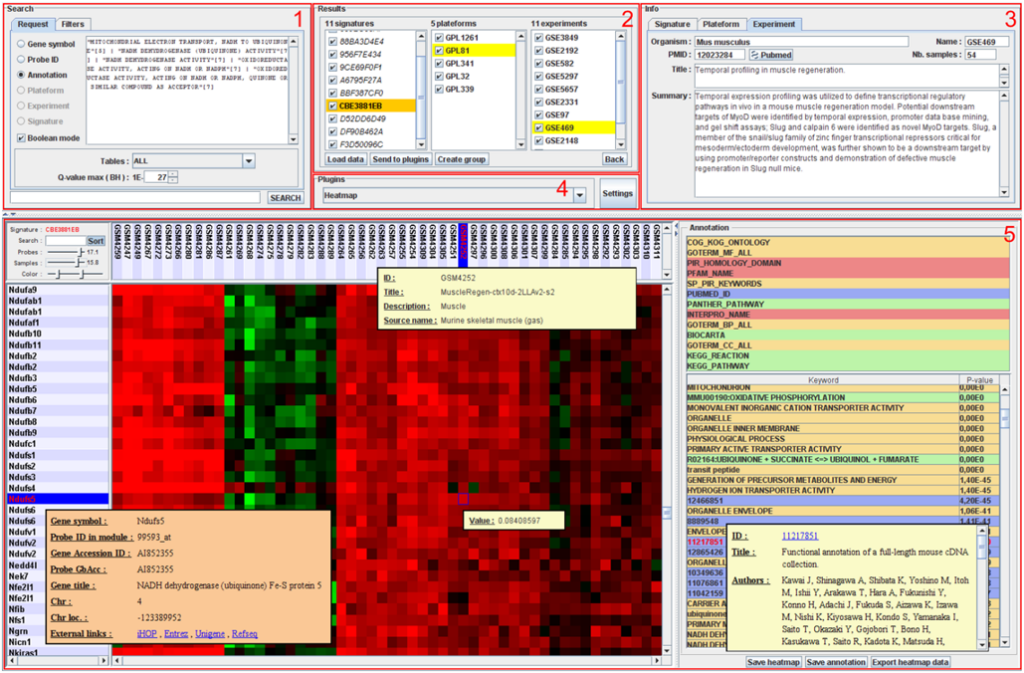
The TBrowser 2.0 interface. The main window of TBrowser is made of five panels (highlighted in red): the search panel (1), the results panel (2), the information panel (3), the plugins panel (4) and the plugin display panel (5). This example shows the expression profiles of genes contained in the TS CBE3881EB derived from GSE469 (“Temporal profiling in muscle regeneration”). Annotation panel shows that this TS is highly enriched in genes related to ATP synthesis.

Comprehensive information on samples, experiments, probes and genes were stored in a mySQL relational database. A flat file indexed on TS IDs was used to store TS expression data. This solution was preferred because it turned out to be an excellent alternative to database for retrieving rapidly expression values for the selected TS. We next developed TBrowser, a Multitier architecture system composed of (i) a “heavy client” written in JAVA (presentation Tier), (ii) a servlet container (logic tier) and (iii) a back-end database (data tier). The client application allows user to query TBrowser database using six methods: by gene symbols, by probe IDs, by experiments, by microarray platform, by ontology terms (annotation) or by TS. Three of them (gene symbols, probe IDs, and annotation methods) accept a list of operators that control the way a query is to be processed. One may take advantage of these operators to create complex queries using the AND operator (&), the OR operator (|), the NOT operator (!) or using additional characters such as the quote or parenthesis (reader may refer to the user guide for additional explanations and informations). The main window of TBrowser is made of five panels (Fig. 3). The search panel is the main entry as it is used (i) to define the search method, (ii) to write the queries, (iii) to launch database interrogation and (iv) eventually to filter out some of the TS. Filters can be applied to select species of interest and to control the sizes (number of samples and number of genes) of the TS that one wants to analyze. The results area can display two panels: the list of queries the user launched during his session and the list of TS that correspond to the currently selected query. Double-clicking on one (or several) TS send it (them) to the selected plugin. The information area is used to display various informations about the selected TS whereas the plugin area is used to select one of the currently installed plugins. Finally, the plugin display panel manages the display of the currently selected plugin. To date, eight plugins have been developed (three of them are presented in this article). The Heatmap plugin is composed of two main panels: the heatmap on the left and the annotation panel on the right (Fig. 3). The Heatmap panel displays a color-coded image of TS expression values. In this representation, each row corresponds to a probe and each column to a sample. Additional informations, such as external links, can be retrieved by single-click on genes or samples. Functional enrichment informations are available on the right. The TBCommonGenes plugin was developed to compare gene composition of several TS and will be presented in the next section. Finally the TBMap plugin that can be used to visualize a summary of transcriptional regulation events observed in a given microarray platform will be presented in the last paragraph of the results section.

### Meta-analysis of public microarray data using TBrowser: a case study

TBrowser can be used in many biological contexts to point out relevant experiments and construct robust gene networks. Several peer-reviewed publications have highlighted the joint regulation of the estrogen receptor-a (ESR1/ER-α), GATA3 and FOXA1 in breast cancer cells [14]. Although some of these reports have associated entry in the GEO database, retrieving neighbors of GATA3, FOXA1 and ESR1 remains a time consuming and difficult task using existing tools. As a consequence, these informations are reserved to those with strong bioinformatics skills although they are of primary interest to the biologist. Using the TBrowser search engine, this task can be translated into a very simple Boolean query, “ESR1 & GATA3 & FOXA1”, which will be almost instantaneously proceeded by the server. With the current database release, this produces a list of 16 TS (see Table 1) containing on average 508 probes (range: 82–1,572) and which were obtained using various microarray platforms (GPL96, GPL570, GPL91). Interestingly, all these TS are related to experiments performed on breast cancer cells underlying the high specificity of this gene list (Table 1). The TBCommonGenes plugin indicates that in addition to ESR1, GATA3 and FOXA1 two genes (ANXA9 and ERBB4) are found in all 16 TS. Importantly, 63 genes are found in at least 10 out of the 16 selected TS (63%). As expected, this list contains numerous markers of breast cancer cells whose expression specificity was previously reported by other (notably ERBB3, XBP1, KRT18, IL6ST, CREB1, TFF1, TFF3; see Supplementary Table S3). Thus TBrowser can be used to perform meta-analysis of microarray data in a platform-independent manner providing high confidence gene lists. However, one can also focus the analysis on a unique platform. Indeed, the transcriptional signatures 3DE64836D, B79B1C0B9 and E2E620F40 that were derived from the GPL570 platform (which measures over 47,000 transcripts) share a list of 68 genes. Many of them correspond to poorly characterized genes (for example, C17orf28 C1orf64, KIAA1370, KIAA1467, LOC143381, LOC400451, LOC92497 and ZNF703). This example clearly demonstrates the superiority of TBrowser over conventional approaches as it can be used, easily and productively, to create robust sets of transcriptionally related genes whose subsequent analysis may be crucial in defining new therapeutic targets.

**Table 1:** Transcriptional signatures containing Affymetrix probes for ESR1, GATA3 and FOXA1.

TS ID^1^	Genes^2^	Probes^2^	Samples^2^	Sample type	GSE ID	GPL ID	Author	PubMed IDs
0F2635383	1190	1572	23	Cell lines	GSE6569	GPL96	Huang F et al 2007	17332353
3DE64836D	102	143	62	Tissue	GSE7904	GPL570	unpublished 2007	-
59A18E225	690	893	121	Both	GSE2603	GPL96	Minn AJ et al 2005	16049480
6C975B20B	88	96	26	Tissue	GSE6772	GPL96	Klein A et al 2007	17410534
6C975B290	88	96	26	Tissue	GSE6596	GPL96	Klein A et al 2007	17410534
7150E17F6	868	1032	34	Cell lines	GSE4668	GPL96	Coser KR et al 2003	14610279
8059848B4	200	250	251	Tissue	GSE3494	GPL96	Miller LD et al 2005	16141321
84E5E1077	694	883	198	Tissue	GSE7390	GPL96	Desmedt C et al 2007	17545524
8F69864F9	68	82	95	Tissue	GSE5847	GPL96	Boersma BJ et al 2007	17999412
A151D5695	297	361	58	Tissue	GSE5327	GPL96	Minn AJ et al 2007	17420468
B79B1C0B9	270	380	47	Tissue	GSE3744	GPL570	Richardson AL et al 2006	16473279
BDB6D8700	550	679	104	Tissue	GSE3726	GPL96	Chowdary D et al 2006	16436632
D8F0B528C	125	152	159	Tissue	GSE1456	GPL96	Pawitan Y et al 2005	16280042
E2E620F40	448	616	129	Tissue	GSE5460	GPL570	unpublished 2007	-
EA9669A21	219	251	158	Tissue	GSE3143	GPL91	Bild AH et al 2006	16273092
F310ACC36	519	646	49	Tissue	GSE1561	GPL96	Farmer P et al 2005	15897907

1 Transcriptional signature ID.

2 Total number.

### Using annotation terms to mine public microarray data

Based on the systematic functional enrichment analysis, the vast majority of TS (84%) have a set of associated biological terms (only functional enrichment with q-value<0.01 are stored in the database). One can search for TS related to functional terms of the DAVID knowledgebase (*e.g.* “nervous system development”). More interestingly, multiple terms can be combined with Boolean operators. Searching for TS which contain genes located in the 6p21.3 and 14q32.33 chromosomal regions (major histocompatibility complex and human immunoglobulin heavy-chain locus respectively) and which contain T-cell specific genes, can be translated as: 6p21.3[4] & 14q32.33[4] & “T CELL ACTIVATION”[5], [12] ([4] = cytoband term, [5] = GO term, [12] = Panther pathways term). As chromosomal aberrations do occur frequently in cancer our approach can also be used to perform systematic cytogenetic analysis. Indeed, throughout our analysis, 2,208 functional enrichments related to 360 human cytobands were observed and stored in the database. As an example, TS with very strong enrichment (q-value<1.10^−20^) for any of the human cytobands stored in the database are presented in Table 2. The first one is related to atopic dermatis analysis (skin biopsies) and contained 24% of genes located in 17q12-q21. They correspond to genes encoding for the keratin and keratin-associated protein families (KRT17, KRT27, KRTAP1-5, KRTAP17-1, KRTAP3-1, KRTAP3-3, KRTAP4-10, KRTAP4-12, KRTAP4-13, KRTAP4-15, KRTAP4-2, KRTAP4-3, KRTAP4-5, KRTAP4-8, KRTAP4-9, KRTAP9-2, KRTAP9-3, KRTAP9-4 and KRTAP9-8). This signature is notably annotated as being enriched in genes related to PMID 11279113 (“Characterization of a cluster of human high/ultrahigh sulfur keratin-associated protein genes embedded in the type I keratin gene domain on chromosome 17q12-21”) [15] and in genes related to the PIR keyword “multigene family”. Furthermore, several signatures, of Table 2 are related to melanoma and six of them were observed in the GSE7127 experiment [16]. Although data from Table 2 would deserve further analysis they are most likely related to gain or loss of genetic material in tumors. Indeed, gain of 8q is frequently observed in a number of tumor types (including melanoma and ovarian tumors) and this region is known to contain the c-myc oncogene at 8q24.21. Interestingly, in several cases, contiguous cytobands were significantly enriched suggesting a large deletion or amplification of genetic material in these tumors (TS 60E29DA83 is enriched in genes from 8q13, 8q21.11, 8q22.1, 8q22.3, 8q24.13 and 8q24.3 cytobands). In the same way, loss of genetic material of the long arm of chromosome 11 occurs in primary melanoma but is even more frequent in metastatic tumors (TS A93ED7519 is enriched in genes from 11q21, 11q23.3 and 11q24.2 cytobands). Altogether, these results underline the versatility of TBrowser and its ability to extract hidden and meaningful informations from published or unpublished microarray data. Indeed, the cytogenetic results presented in Table 2 were not discussed by the authors in the corresponding articles.

**Table 2:** Transcriptionnal signatures displaying high enrichment (q value<1.10−20 ) for any of the human cytoband tested.

TS ID^1^	Enrich.^2^	Cytoband	q.value	Sample type	GSE ID	GPL ID	Authors	PubMed ID
3DA3C8345	24%	17q12-q21	1.7.10^−39^	Skin	GSE5667	GPL97	Plager DA et al 2007	17181634
43CC3EF57	9%	8q24.3	7.0.10^−32^	Melanoma	GSE7153	GPL570	Unpublished 2007	-
60E29DA83	16%	8q24.3	6.8.10^−24^	Melanoma	GSE7127	GPL570	Johansson P et al 2007	17516929
60E581184	26%	17q25.1	5.5.10^−23^	Melanoma	GSE7127	GPL570	Johansson P et al 2007	17516929
60E6B4129	35%	20p13	1.6.10^−26^	Melanoma	GSE7127	GPL570	Johansson P et al 2007	17516929
60E96FF1E	28%	6p21.3	1.2.10^−28^	Melanoma	GSE7127	GPL570	Johansson P et al 2007	17516929
60EC95F6A	17%	7q22.1	6.3.10^−31^	Melanoma	GSE7127	GPL570	Johansson P et al 2007	17516929
60EEBD669	32%	11q23.3	1.4.10^−26^	Melanoma	GSE7127	GPL570	Johansson P et al 2007	17516929
B4C95CF18	42%	8q24.3	1.1.10^−36^	Ovary	GSE6008	GPL96	Hendrix ND et al 2006	16452189
A93ED6519	16%	11q23.3	6.9.10^−23^	Melanoma	GSE7152	GPL570	Packer LM et al 2007	17450523
A93DB01ED	11%	7q22.1	9.5.10^−30^	Melanoma	GSE7152	GPL570	Packer LM et al 2007	17450523

1 Transcriptional signature ID.

2 Enrichment: Proportion of non redondant genes from the TS that are located in the corresponding cytoband.

### A synthetic view of all GPL96 related experiments

**Figure 4:**
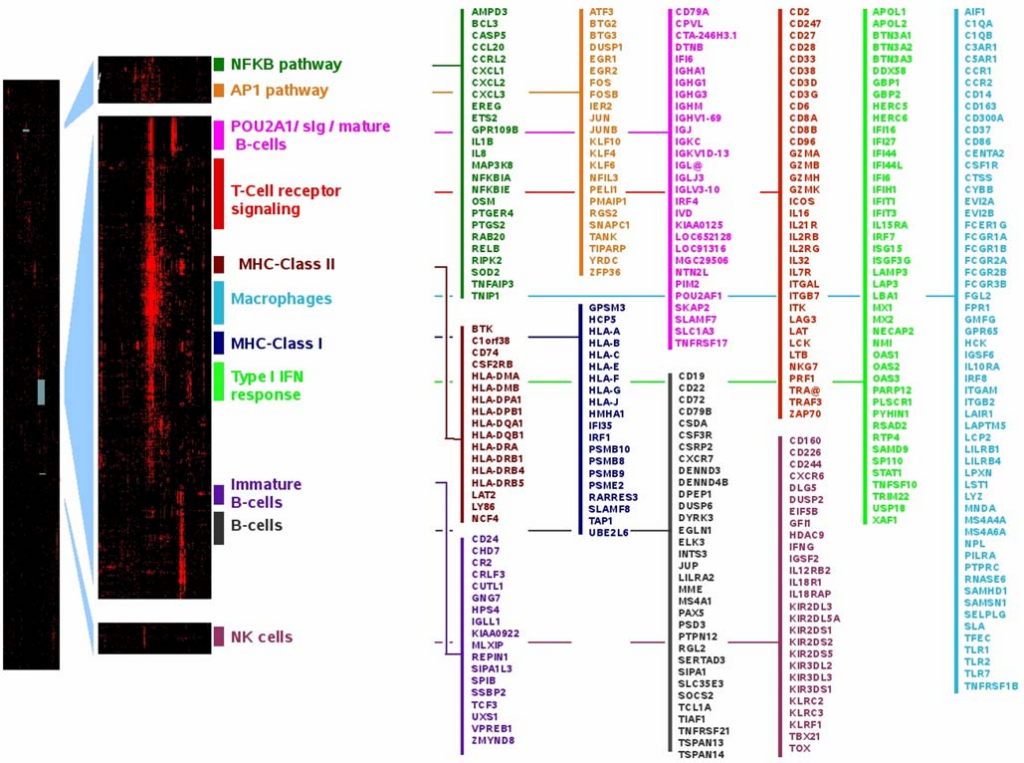
The transcriptional MAP associated with GPL96 related experiments. (A) A low resolution image made of 22,215 probes from GPL96 platform as rows and 3,114 GPL96 specific TS as columns. Red color indicates the presence of a gene in the corresponding TS (default to black). (B) Zooms of the corresponding areas showing some immune system related meta-signatures. (C) Representative genes that fall into these clusters.

The paradigm that genes from a TS share functional relationships is now widely accepted and constitutes the basis of transcriptome analysis [17]. However, each of these TS is rather associated to multiple underlying pathways whose components and limits are unclear. Our difficulty in depicting comprehensive maps for pathways is illustrated by existing discrepancies, for instance, between those proposed by BioCarta, KEGG and GeneMAPP. We reasoned that the more frequently two genes fall in the same TS, the more likely these genes belong to the same core functional network. To test this hypothesis, we produced a Boolean matrix with 22,215 probes from GPL96 platform as rows and 3,114 GPL96 specific TS as columns (only TS containing 30 to 1500 probes were included). This matrix was filled with zero and elements were set to 1 if a given gene was observed in the corresponding TS. Hierarchical clustering with uncentered Pearson's correlation coefficient was used to reveal genes frequently associated to the same TS. Given the order of the resulting matrix, it could not be visualized on a desktop computer using conventional software (*i.e.*; Treeview, MeV). We thus developed the TBMap plugin which allows one to visualize the map but also to superimpose a user-defined or a KEGG-related gene list. As expected, most of the clusters where obviously enriched in genes involved in similar biological processes (Protein biosynthesis/Ribosome function, oxidative phosphorylation, cell cycle, fatty acid metabolism, valine leucine and isoleucine degradation, extracellular matrix, breast cancer cells, structural constituent of muscles, neuronal processes, etc.). This was particularly clear when KEGG pathway informations were superimposed (see Figure S6). The Figure 4 presents some of the clusters that were identified as related to immune system functions. We could find a signature defining T cells that contained numerous cell-surface markers (e.g. TCA@, CD2, CD3G, CD6, IL2RB, IL2RG, IL7R, IL21R and ICOS), signaling genes (ZAP70, LAT, LCK, ITK) and cytotoxicity-related genes (GZMA, GZMB, GZMH, GZMK and PRF1). Concerning B-cells, three clusters were observed. A large signature contains mature B-cell markers (CD19, CD22, CD72 and CD79B) and transcription factors important in B-cell development such as PAX5 and TCL1A. A second signature contains POU2AF1/OBF-1, together with its described targets: genes coding for immunoglobulin (IGHG1, IGHG3, IGHA1, IGHM, IGJ, IGKC and IGL) and the B-cell maturation factor, TNFRSF17/BCMA [18], [19]. The third B-cell signature contains cell surface markers found in immature B-cells (CD24, VPREB1, IGLL1/CD179B and CR2/CD21) in addition to transcription factors known to play a crucial role during early B-cell development (TCF3, SPIB and CUTL1). The NK signature contains eight genes of the Killer cell immunoglobulin-like receptors (KIR) family, 3 genes of the killer cell lectin-like receptor family in addition to other markers whose expression has been reported on the surface of NK cells (CD160, CD244/2B4 and CD226) [20], [21], [22]. It also contains TBX21/T-bet together with IL18R1, IL18RAP, IL12RB2 and IFNG. Importantly, the IL12/IL18 combination has been shown to be potent inducers of both TBX21/T-bet and IFNG in NK cells[23], [24]. In addition to MHC-Class I, MHC-Class II and macrophage related signatures, two pathways related to immune function are presented in Figure 4. The AP1 pathway is made of the prototypical immediate early genes and contains numerous transcription factors (EGR1, EGR2, FOS, FOSB, IER2, JUN, JUNB, KLF6, KLF4, KLF10, ATF3, BTG2 and BTG3) whose complex interplay has been reported earlier. Finally, a NFKB signature was also observed which, again, contains prototypical regulators (NFKIA, NFKIE, RELB, BCL-3 and MAP3K8/TPL2) and known targets (CCL20, CXCL3, IL1B, IL8 and SOD2). Altogether, these results underline the high relevance of the signatures obtained using this compilation of TS derived from GPL96 related GEO experiments.

## Discussion

In the present paper, we present the construction of a unique collection of TS that summarize almost all human, mouse and rat Affymetrix microarray data stored in the GEO database. TBrowser constitutes a highly powerful search engine that makes it possible to perform easily platform independent meta-analysis of microarray data. This can be considered as a real improvement over classical approaches and softwares as it provides easy and productive access to data without the need of any programming skills. Indeed the simple use of an extended set of operators proved to be sufficient to construct robust gene networks and assign poorly characterized genes to relevant biological pathways. As a consequence, it is particularly well suited to compare results obtained through microarray, ChIP-on-chip, ChIP-seq, CGH or protein-protein interaction experiments to those previously stored in the GEO database.

In all tested experiments, we found that DBF-MCL gives very good results both on simulated datasets and real microarray datasets. Although Lattimore *et al* proposed another MCL-based algorithm (geneMCL) we were unable to compare our results with their implementation as the software is no longer available nor maintained. However, DBF-MCL was run on the full van't Veer DataSet [25] (117 biological samples) that was used by Lattimore and collaborators in the original paper. In their report, the authors used a subset of genes (5,730 out of 24,482) that were selected based on their associated variance. Our procedure run on the full dataset led to the selection of 5,932 genes that fall into 22 clusters (in contrast to 154 clusters using geneMCL). This discrepancy is likely to be due to the filtering step applied to the dataset. Indeed, a strong associated variance can also be reminiscent of punctual random artifacts. Thus, selecting those genes will generate small or singleton clusters. In this context, the MDNN statistic better handle these artifacts as its purpose is to conserve genes that belong to dense region in the hyperspace.

To date, TBrowser provides user with only one partitioning solution for a dataset. However as density is heterogeneous inside a dataset, several partitioning solutions exist. For instance, if one observes a cluster containing cells of the immune system this will also frequently contain several sub-clusters that will be reminiscent of cell types (B- or T-cells for example) or activation status. Increasing MCL granularity (“Inflation” parameter) will most generally split the parent clusters and provided user with another partitioning result. However, both results can be considered as optimal and we should consider all of them. To this end we plan to propose multiple partitioning solutions for each dataset to provide a more exhaustive view of underlying biological pathways. Although, such an approach could appear computer-intensive it should be practicable, taking into account that DBF-MCL is much faster than hierarchical clustering or MCL run on a whole dataset. In addition, although we routinely obtained very relevant results with DBF-MCL, we expect that even more accurate methods will be proposed in the future.

The present work focus on human, mouse and rat Affymetrix microarray data but TBrowser can handle any type of microarrays and organism. The current release of the database already contains data obtained using other commercial (*e.g.* Agilent, Illumina Inc., GE Healthcare, Applied Biosystems, Panomics, CapitalBio Corporation, TeleChem ArrayIt, Mergen-LTD, Eppendorf Array Technologies) and non commercial platforms (*e.g.* National Cancer Institute, Vanderbilt Microarray Shared Resource, Genome Institute of Singapore), several of them being related to the MicroArray Quality Control (MAQC) project (GSE5350) [26]. However, to date, systematic analysis of all experiments performed on these platforms has not been done. The flexibility of our approach also makes it possible to integrate and compare data obtained through any kind of large scale analysis technologies providing that the experiment can be represented by a single numerical matrix (ChIP-on-chip, Protein array, large scale Real-time PCR, ChIP-seq, etc.). Three plugins (Heatmap, TBCommonGenes and TBMap) have been presented in this article but seven new plugins have been recently developed (manuscript in preparation). In the near future, the ease of plugin development will makes it possible to look for TS enriched in genes sharing transcription factor and miRNA specific motifs in their non-coding regions.

As raw data are only available for some of the microarray datasets, we used the “normalized” data provided by submitters. These data were subsequently rank-transformed and used for classification. This procedure allowed us to re-analyze a very large number of datasets. However, the drawback is that quality status of individual samples or experiments could not be determined (computing the so-called “3′/5′ ratio” requires raw data). We plan to provide extensive quality control informations through a dedicated plugin. However, we think that scientists should comply better with the MIAME guidelines and that they should provide systematically raw data when submitting a new experiment. Finally, we would like to acknowledge the GEO database team whose efforts in providing high quality repository service made this work possible.

## Materials and Methods

### Microarray data retrieval

Human mouse and rat microarray data derived from 30 Affymetrix microarray platforms (Supplementary Table S1) were downloaded from the GEO ftp site and retrieved in seriesMatrix file format (ftp://ftp.ncbi.nih.gov/pub/geo/DATA/SeriesMatrix/). SeriesMatrix are summary text files related to a GEO series Experiment (GSE) that include sample and experiment metadata together with a tab-delimited matrix that corresponds to normalized expression data. Each file (n = 2,869) was parsed using a Perl script to extract gene expression matrix and metadata. Probes with missing expression values were excluded from analysis. Only expression matrix with at least ten columns/samples were kept for subsequent analysis (n = 1,484, Supplementary Table S2).

### DBF-MCL algorithm

The filtering step of DBF-MCL was implemented in C. The latest Markov Clustering algorithm version (1.006, 06-058) was obtained from http://micans.org/mcl/src/. The full pipeline of DBF-MCL (that integrates normalization, filtering and partitioning) was implemented in Bash Shell Scripting language. This script supports different metrics for distance calculation (Euclidean distance, Pearson's correlation coefficient-based distance, Spearman's rank correlation-based distance).

### Data normalization and processing

Given the huge amount of data processed by GEO curators it is impractical to determine the quality and efficiency of the normalization methods used [27]. Although seriesMatrix files should ideally contain log-transformed data, expression matrices in linear scale were also observed in several cases. To circumvent this problem each column of the expression matrix was rank-transform (using R software). This normalization procedure is insensitive to data distribution and provided us with a standard input for the DBF-MCL algorithm. In the case of microarray data, DBF-MCL was run using Spearman's rank correlation-based distance (1-r). However, although rank-based methods are well suited for normalization and distance calculation purposes they are not appropriate to display gene expression profiles. To this end, a normal score transformation was applied to each column of the datasets after DBF-MCL classification. The transformation ensures that whatever the data a standard format is available for heatmap visualization. Finally, for each experiment, this dataset was used (1) to classify samples using hierarchical clustering (2) to build the expression matrix for the corresponding TS.

### Data storage

Expression matrix for each TS were stored in an indexed flat file with a TS ID as a key. This flat file is used by the TBrowser client to retrieve expression data for the requested TS. Experiment metadata, corresponding to sample and experiment informations were stored in a mySQL relational database. Probe meta-information (gene symbol, gene name, GenBank accession ID, chromosomal location, Entrez ID) were obtained from Bioconductor [28] annotation packages and stored in the database. In some cases, as no annotation packages were available (especially for GeneChip® CustomExpress® Array) a script was used to obtain gene symbols and gene names from GenBank files based on the provided GenBank accession ID. Both flat file and database information will be periodically updated to give access to novel experiments stored in GEO repository.

### Complex9 dataset

The complexe9 dataset was obtained from the UH Data Mining and Machine Learning Group (UH-DMML, http://www2.cs.uh.edu/~ml_kdd/). Cluster Affinity Search Technique (CAST) was run using the TMEV software. QT_CLUST and k-means were run using the flexclust and fpc R package. For k-means, the algorithm was run 10 times with random initial centers. Hierarchical clustering was performed using the amap library from the R/Bioconductor project. The Euclidean distance was used in all cases.

### Functional enrichment analysis

We used the DAVID knowledgebase [12] for functional enrichment analysis as it provided a practical mean to gain access to a wide range of heterogeneous sources of gene annotation (152,543 annotation terms were used for human, 105,207 for mouse and 39,787 for rat). DAVID ID mapping was obtained for 218,727 AffyID. A Perl script that integrates call to the R software was run to load probe list and calculate iteratively Fisher's exact test p-values on 2×2 contingency tables. Bonferroni adjusted *p*-values were calculated using the multtest Bioconductor library for all TS. Overall, 5.10^6^ Fisher's exact test were performed.

### User interface

TBrowser is accessible through a web browser at TAGC web site (http://tagc.univ-mrs.fr/tbrowser/). Of note, the TBrowser client is extensible through a plug-in architecture that allows rapid development of additional features. A developer's guide will be available soon on our website.

## Supporting Information

Figure S1 A schematic overview of the pipeline used in TBrowser.
(10.16 MB TIF)

Figure S2 An illustration in two dimensions of the motivation behind DBF-MCL filtering step. Arrows point out the 20th nearest neighbor for selected points. Length of each segment corresponds to a given DKNN value.
(8.22 MB TIF)

Figure S3 Distributions of DKNN values. Observed DKNN values (solid line) and of a set of simulated DKNN values S (dotted line) are shown for (A) the Complex9RN200 artificial dataset and (B) the GSE1456 microarray dataset.
(9.01 MB TIF)

Figure S4 Colors correspond to the clusters found using the corresponding algorithm (A) The whole dataset (9,112 points). (B) A zoom-in of Complex9RN200 dataset that displays the various shapes to be found. (C) DBF filtering step without partitioning. With k set to 60, noisy elements remain around the shapes. (D–G) The filtering and partitioning results obtained using DBF-MCL run with a range of k values and I values. Other arguments are unchanged (FDR = 10%, S1..3). The set of points (n = 3,108) obtained using DBF-MCL (k = 20) was used to test the other algorithms (H) Results obtained with hierarchical clustering (single linkage). The obtained dendrogram was cut to produce 9 clusters. (I) Results obtained with the QT_CLUST algorithm (radius = 0.8). (J) Results obtained for k-means (9 centers, 100 initializations). (K) Results obtained with cst(threshold = 0.81).
(9.41 MB TIF)

Figure S5 Impact of various k values on DBF-MCL results. The x-axis correspond to k values. The y-axis correspond to the number of elements considered as informative. (A) DBF-MCL was run with the Complex9RN200 as input using a range of k values (FDR = 10%, S1..3, Inflation = 1.2). (B) DBF-MCL was run with several microarray datasets as input (including GSE1456) using a range of k values (FDR = 10%, S1..3, Inflation = 2).
(8.72 MB TIF)

Figure S6 The TBMap plugin. These pictures are derived from the GPL96 map (22,215 probes as rows and 3,114 GPL96 specific TS as columns). Red indicates the presence of a gene in the corresponding TS (default to black). Only small parts of the map are displayed. (A) A cluster enriched in genes from the “Aminoacyl-tRNA biosynthesis” KEGG pathway (hsa00970). Genes (rows) from this KEGG pathway are displayed as blue lines (CARS, SARS, AARS, GARS, MARS, IARS, YARS). Genes from a manually entered gene list are shown in yellow (TRIB3, MOCOS, MPZL1, CBS, PPCDC). (B) A cluster enriched in genes related to oxydative phosphorylation (KEGG pathway hsa00190, “Oxidative phosphorylation”). (C) A cluster containing genes related to ribosome biogenesis (KEGG pathway hsa03010 “Ribosome”). (D) A cluster enriched in genes involved in cell proliferation (KEGG pathway hsa04110 “Cell cycle”).
(9.66 MB TIF)

Table S1 Informations related to Affymetrix platforms (n = 33) used in the present work.
(0.12 MB XLS)

Table S2 Informations related to experiments (n = 1,484) that were analyzed using the DBF-MCL algorithm. All Informations were obtained from the GEO website.
(1.38 MB XLS)

Table S3 This matrix summarizes the results obtained using the “ESR1 & GATA3 & FOXA1” query. Rows correspond to genes and columns to TS. The presence of a given gene in a given TS is indicated by 1 (default 0).
(0.66 MB XLS)
