# Supplementary figures and images for: TranscriptomeBrowser: A Powerful and Flexible Toolbox to Explore Productively the Transcriptional Landscape of the Gene Expression Omnibus Database
Source: PLoS One. 2008 Dec 23;3(12):e4001. doi: 10.1371/journal.pone.0004001 (PMC2602602; doi:10.1371/journal.pone.0004001)

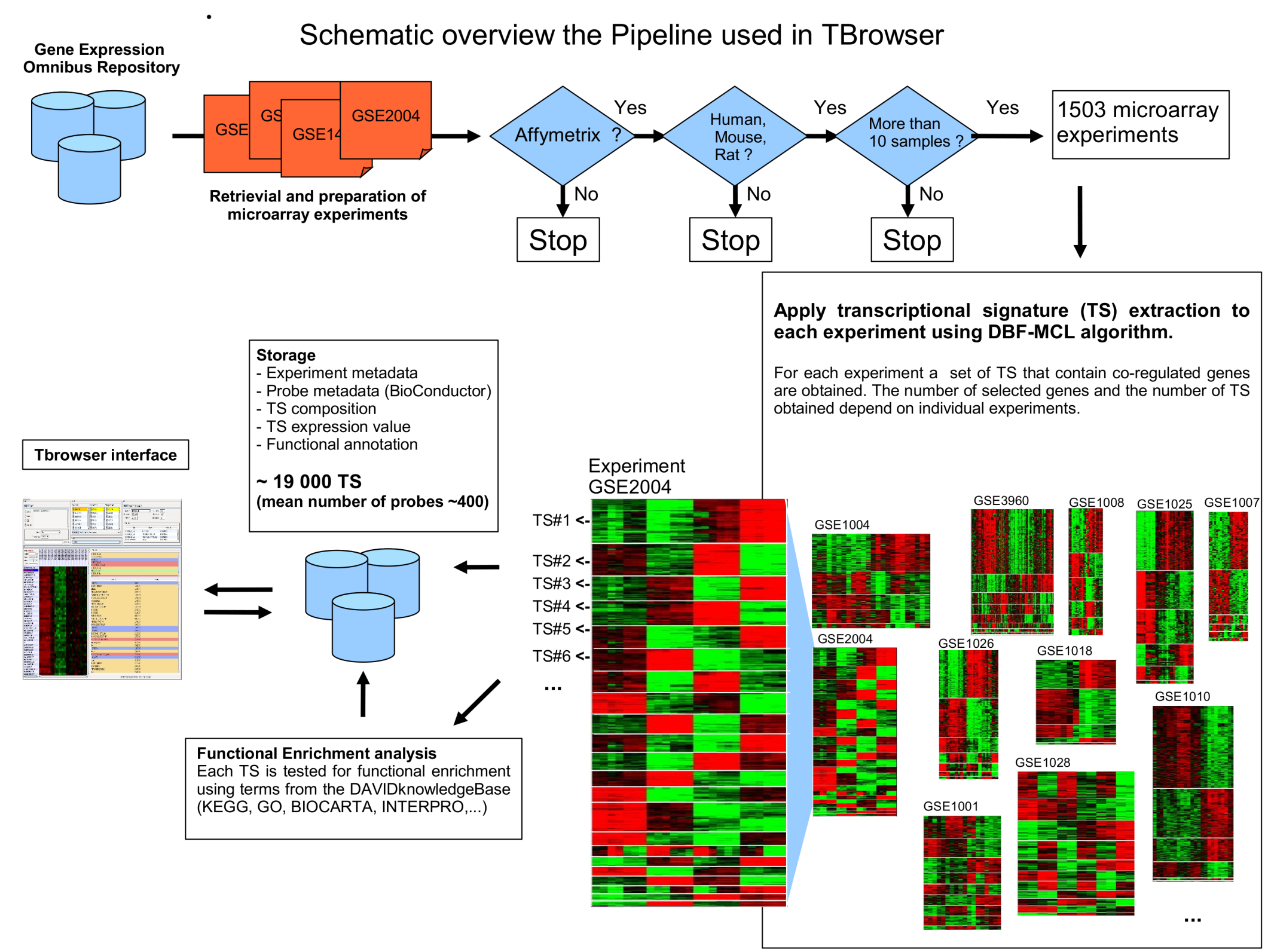

Supplement: Figure S1 — A schematic overview of the pipeline used in TBrowser. (10.16 MB TIF) [file pone.0004001.s001.tif]

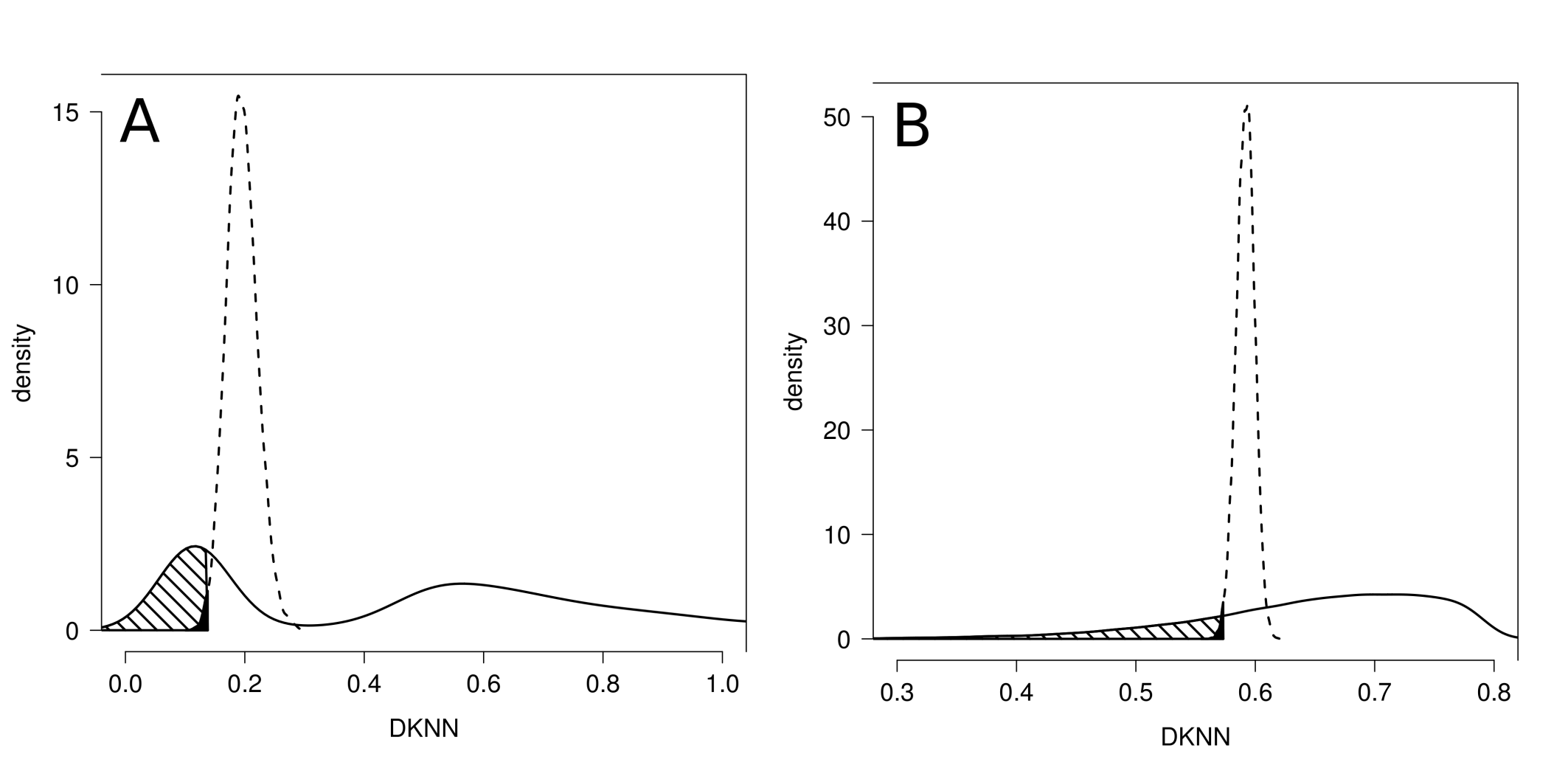

Supplement: Figure S3 — Distributions of DKNN values. Observed DKNN values (solid line) and of a set of simulated DKNN values S (dotted line) are shown for (A) the Complex9RN200 artificial dataset and (B) the GSE1456 microarray dataset. (9.01 MB TIF) [file pone.0004001.s003.tif]

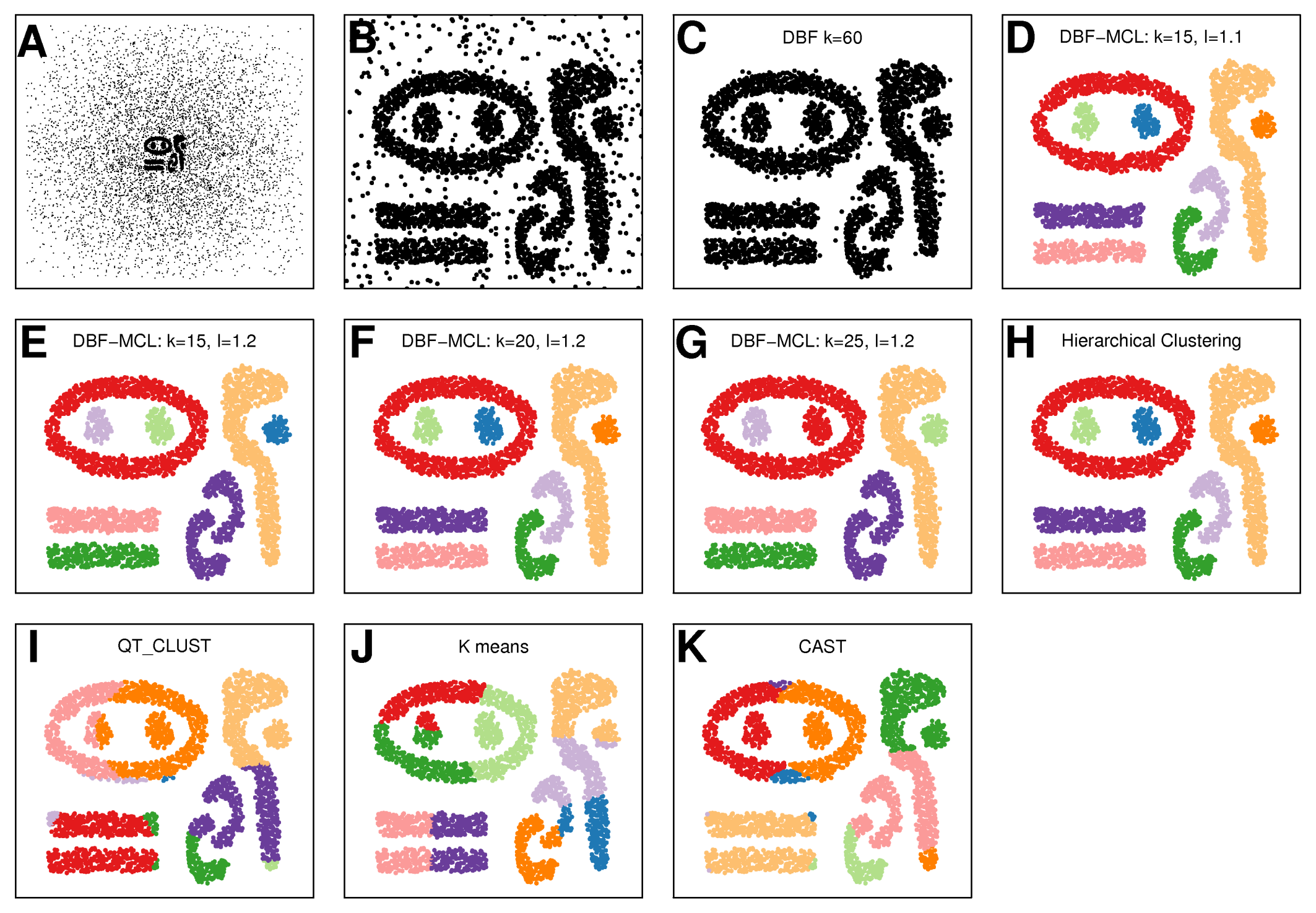

Supplement: Figure S4 — Colors correspond to the clusters found using the corresponding algorithm (A) The whole dataset (9,112 points). (B) A zoom-in of Complex9RN200 dataset that displays the various shapes to be found. (C) DBF filtering step without partitioning. With k set to 60, noisy elements remain around the shapes. (D–G) The filtering and partitioning results obtained using DBF-MCL run with a range of k values and I values. Other arguments are unchanged (FDR = 10%, S1..3). The set of points (n = 3,108) obtained using DBF-MCL (k = 20) was used to test the other algorithms (H) Results obtained with hierarchical clustering (single linkage). The obtained dendrogram was cut to produce 9 clusters. (I) Results obtained with the QT_CLUST algorithm (radius = 0.8). (J) Results obtained for k-means (9 centers, 100 initializations). (K) Results obtained with cst(threshold = 0.81). (9.41 MB TIF) [file pone.0004001.s004.tif]

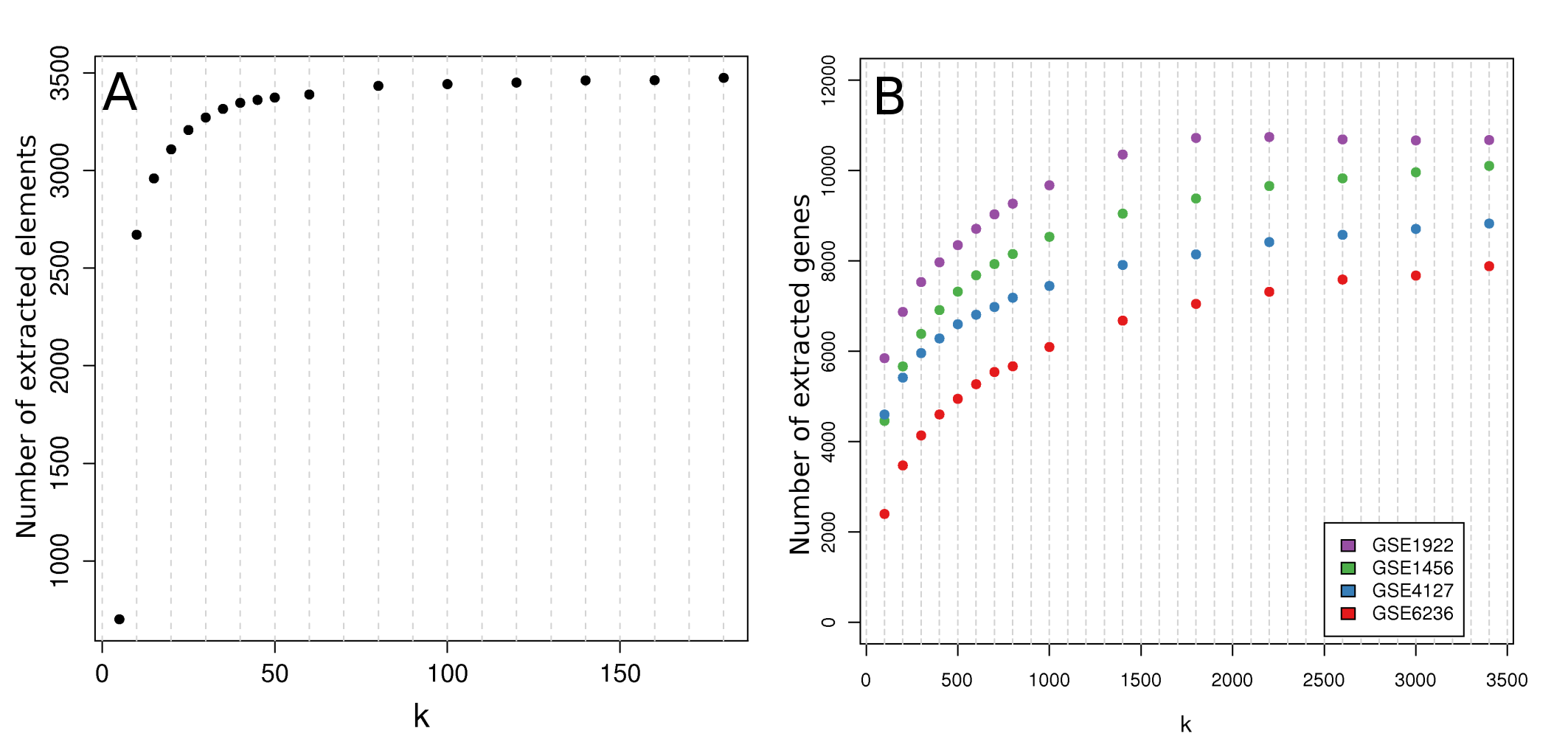

Supplement: Figure S5 — Impact of various k values on DBF-MCL results. The x-axis correspond to k values. The y-axis correspond to the number of elements considered as informative. (A) DBF-MCL was run with the Complex9RN200 as input using a range of k values (FDR = 10%, S1..3, Inflation = 1.2). (B) DBF-MCL was run with several microarray datasets as input (including GSE1456) using a range of k values (FDR = 10%, S1..3, Inflation = 2). (8.72 MB TIF) [file pone.0004001.s005.tif]

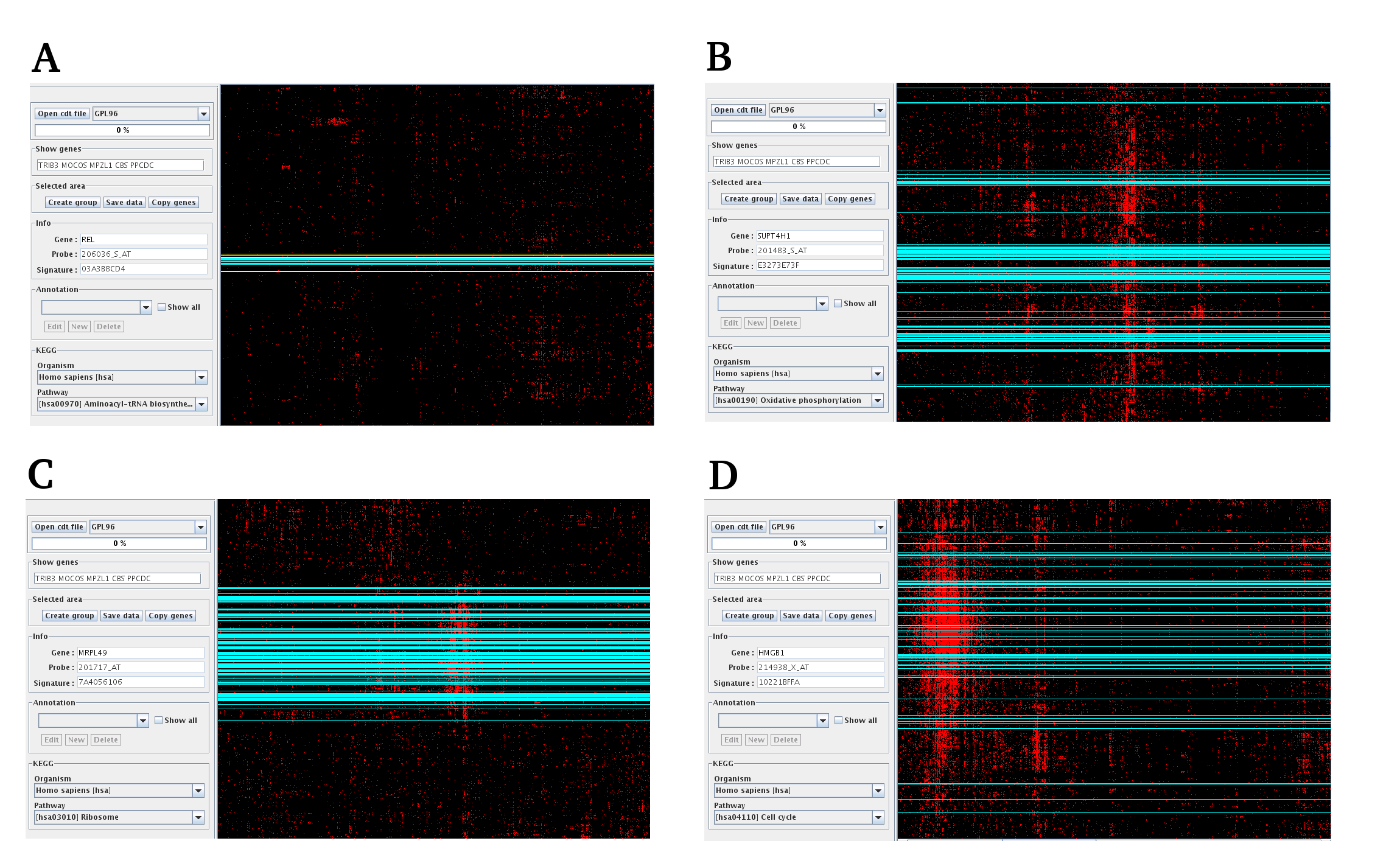

Supplement: Figure S6 — The TBMap plugin. These pictures are derived from the GPL96 map (22,215 probes as rows and 3,114 GPL96 specific TS as columns). Red indicates the presence of a gene in the corresponding TS (default to black). Only small parts of the map are displayed. (A) A cluster enriched in genes from the “Aminoacyl-tRNA biosynthesis” KEGG pathway (hsa00970). Genes (rows) from this KEGG pathway are displayed as blue lines (CARS, SARS, AARS, GARS, MARS, IARS, YARS). Genes from a manually entered gene list are shown in yellow (TRIB3, MOCOS, MPZL1, CBS, PPCDC). (B) A cluster enriched in genes related to oxydative phosphorylation (KEGG pathway hsa00190, “Oxidative phosphorylation”). (C) A cluster containing genes related to ribosome biogenesis (KEGG pathway hsa03010 “Ribosome”). (D) A cluster enriched in genes involved in cell proliferation (KEGG pathway hsa04110 “Cell cycle”). (9.66 MB TIF) [file pone.0004001.s006.tif]
